# Improving cooperation between general practitioners and dermatologists via telemedicine: study protocol of the cluster-randomized controlled TeleDerm study

**DOI:** 10.1186/s13063-018-2955-2

**Published:** 2018-10-24

**Authors:** Roland Koch, Andreas Polanc, Hannah Haumann, Gudula Kirtschig, Peter Martus, Christian Thies, Leonie Sundmacher, Carmen Gaa, Leonard Witkamp, TeleDerm Study Group, Oliver Bertram, Lucien Clin, Sven Dörflinger, Claus Garbe, Thomas Eigentler, Heidrun Sturm, Janina Schubert, Tonia Brand, Katrin Tomaschko, Hans Leibfritz, Aaron Bakker, Job van der Heijden, Julia Frank-Tewaag, Matthias Wöhr, Anika Meissner, Matthias Möhrle, Sebastian Kauder, Markus Krug, Andreas Blum, Stefanie Joos

**Affiliations:** 1 University Hospital Tübingen, Institute for General Practice and Interprofessional Care, Osianderstraße 5, 72076 Tübingen, Germany; 2 AOK Baden-Württemberg Hauptverwaltung, Fachbereich Integriertes Leistungsmanagement, Presselstraße 19, 70191 Stuttgart, Germany; 3 University Hospital Tübingen, Institute for Clinical Epidemiology and Applied Biometry, Silcherstraße 5, 72076 Tübingen, Germany; 4 Reutlingen University, School of Informatics, Alteburgstraße 150, 72762 Reutlingen, Germany; 5 Ludwig-Maximilians-University München, Fachbereich Health Services Management, Schackstraße 4, 80539 Munich, Germany; 6 Academic Medical Centre, Meibergdreef 9, 1105 AZ Amsterdam, the Netherlands; 7 KSYOS TeleMedisch Centrum, Bavinckhouse, Professor J.H. Bavincklaan 2-4, 1183 AT Amstelveen, the Netherlands; 8 Reutlingen University, Reutlingen Research Institute, Alteburgstraße 150, 72762 Reutlingen, Germany; 9 University Hospital Tübingen, Section Dermatologic Oncology, Liebermeisterstraße 25, 72076 Tübingen, Germany; 10 aQua—Institut für angewandte Qualitätsförderung und Forschung im Gesundheitswesen GmbH, Maschmühlenweg 8–10, 37073 Göttingen, Germany; 11 HÄVG Regionaldirektion Süd, Kölner Str. 18, 70376 Stuttgart, Germany; 12 Praxisklinik Tübingen, Gemeinschaftspraxis, Europaplatz 2, 72072 Tübingen, Germany; 13 Hautarzt- und Lehrpraxis Konstanz, Augustinerplatz 7, 78462 Konstanz, Germany

**Keywords:** Telemedicine, Teledermatology, Primary care, Implementation, Referral, Consultation

## Abstract

**Background:**

Internationally, teledermatology has proven to be a viable alternative to conventional physical referrals. Travel cost and referral times are reduced while patient safety is preserved. Especially patients from rural areas benefit from this healthcare innovation. Despite these established facts and positive experiences from EU neighboring countries like the Netherlands or the United Kingdom, Germany has not yet implemented store-and-forward teledermatology in routine care.

**Methods:**

The TeleDerm study will implement and evaluate store-and-forward teledermatology in 50 general practitioner (GP) practices as an alternative to conventional referrals. TeleDerm aims to confirm that the possibility of store-and-forward teledermatology in GP practices is going to lead to a 15% (*n* = 260) reduction in referrals in the intervention arm. The study uses a cluster-randomized controlled trial design. Randomization is planned for the cluster “county”. The main observational unit is the GP practice. Poisson distribution of referrals is assumed. The evaluation of secondary outcomes like acceptance, enablers and barriers uses a mixed-methods design with questionnaires and interviews.

**Discussion:**

Due to the heterogeneity of GP practice organization, patient management software, information technology service providers, GP personal technical affinity and training, we expect several challenges in implementing teledermatology in German GP routine care. Therefore, we plan to recruit 30% more GPs than required by the power calculation. The implementation design and accompanying evaluation is expected to deliver vital insights into the specifics of implementing telemedicine in German routine care.

**Trial registration:**

German Clinical Trials Register, DRKS00012944. Registered prospectively on 31 August 2017.

**Electronic supplementary material:**

The online version of this article (10.1186/s13063-018-2955-2) contains supplementary material, which is available to authorized users.

## Background

Teledermatology is the process of diagnosing dermatologic problems at a physical distance and, in the case of store-and-forward technology, at different times [1]. Internationally, teledermatology is already implemented in a number of healthcare systems [1]. For example, the USA, the Netherlands and the United Kingdom (UK) use teledermatology in clinical routine practice [1–3].

Dermatologic conditions are common in primary care [4–6]. In about 80% of common conditions, the general practitioner (GP) is able to make a diagnosis and initiate treatment based on clinical examination and patient history [7].

If a GP experiences diagnostic uncertainty or if treatment fails, referral to a dermatologist is usually the next step. Physical referrals are often associated with long waiting times for an appointment. Long travel distance between the patient’s home and the dermatologist’s practice is another complication. Thus, teledermatology is a viable alternative to physical referrals—especially in rural areas.

According to previous studies, most common skin diseases can safely be diagnosed by teledermatology [1]. A randomized controlled trial by Whited et al. [8] showed comparable clinical courses and quality of life in 392 patients. The study compared teledermatology with physical referrals to dermatologists. Both patients and care providers evaluated teledermatology positively [8]. Another randomized controlled trial by Eminovic et al. [9] involved 631 patients. The study showed that 21% of all physical referrals to dermatologists could be prevented by teledermatology [9]. Other studies concluded that between 18 and 94% (mean 43%) of physical dermatologist consultations could be prevented by teledermatology [1, 8, 10, 11]. Several studies showed that teledermatology is equal to conventional referrals in terms of accuracy, specificity, sensitivity and clinical endpoints [1, 3, 11–15]. Depending on the health and reimbursement system, teledermatology can lead to cost savings up to 18% compared with conventional referrals [1, 16]. Furthermore, a significant learning effect in GPs was reported. This learning effect is presumed to contribute to a sustainable cost reduction over time [16].

In Germany, however, teledermatology is not yet implemented in routine care. The use of teledermatology in general practice is restricted to local solutions and pilot projects [2, 17]. Specialized first-line dermatologic care in Germany is mainly provided by dermatologists working in their own practices. Only a minority of dermatologists work in hospitals. Thus, access to specialized dermatologic care shows regional differences [18, 19]. Due to the growing number of patients and the relatively difficult access to specialized dermatologic care, GPs will play an increasingly important role in diagnosing and treating dermatologic problems in the future [20]. Demographic change further contributes to the increasing number of skin conditions and malignancies of the skin [4, 20, 21]. Older patients have more difficulties accessing specialized dermatologic care due to reduced mobility [4]. To our knowledge, quite a few patients and GPs in Germany help themselves by using commercial end-to-end encrypted communication platforms such as WhatsApp. Both patients and GPs send clinical data such as dermatologic photographs, radiology images or electrocardiogram (ECG) printouts to specialists to obtain advice. The legality of these actions in the context of rigorous German data protection laws is highly questionable. In summary, this background illustrates the need for telemedicine as an addition to interdisciplinary and cross-sectoral communication in German health care.

Thus, the aim of our study is to evaluate the implementation of teledermatology in primary care in a defined number of counties in Baden-Württemberg, Germany.

## Methods

### Study design and setting

**Fig. 1:**
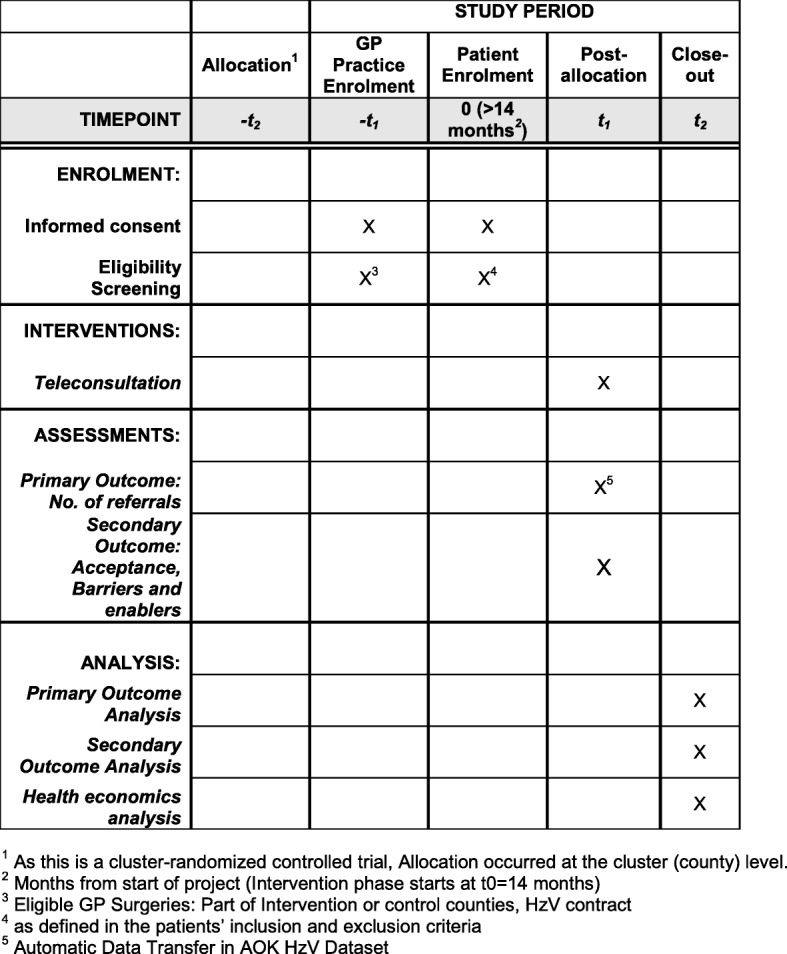
SPIRIT 2013 figure with schedule of enrolment, interventions and assessments. GP general practitioner, HzV Hausarztzentrierte Versorgung

The study is planned as a cluster-randomized controlled confirmatory study, where a county is regarded as one cluster. The main scientific hypothesis is that the possibility of store-and-forward teledermatology in GP practices is going to reduce GP referrals to dermatologists by at least 15%. This protocol follows the Guidance of Standard Protocol Items: Recommendations for Interventional Trials (SPIRIT) 2013 Statement [22]. It includes the schedule of enrolment and relevant assessments (Fig. 1) using the SPIRIT figure template. A SPIRIT checklist is provided in Additional file 1.

This project is one of the first to be supported by the Federal Joint Committee’s Innovation Fund. In order to facilitate innovations in German health care, the fund was created in 2015 with the “Law on Strengthening Statutory Health Care” (Gesetzliche Krankenversicherungs-Versorgungsstärkungsgesetz) [23]. The Federal Joint Committee call for proposals is competitive and uses external peer review. The sponsor has no influence over study design; collection, management, analysis and interpretation of data; writing of the report; and the decision to submit the report for publication. Neither does the sponsor have ultimate authority over any of these actions.

To describe the study setting, a short overview of the health insurance system in Germany is presented. There are around 116 statutory health insurance companies in Germany. The Allgemeine Ortskrankenkasse (AOK) is one of the largest statutory health insurance funds with over 25 million insured persons (35% of all statutory insured persons) [24]. Statutory insurance companies are self-governed corporations under public law. The state of Germany or federal regions may be shareholders in these companies. They must provide health services for everyone who is insured. They also must accept every applicant for health services. Private health insurance companies, on the other hand, are basically privately owned.

In addition to their regular coverage, all health insurance companies may sign direct contracts with GP representatives and other specialist physician groups. An example of such a contract is the Hausarztzentrierte Versorgung (HzV) (GP-centered health care). Patients enrolled in the HzV decide to enlist to one GP. Every primary care physician has regular contracts with the Association of Statutory Health Insurance Physicians (KV). This association negotiates outpatient treatment costs with the legislator and the insurance companies. Within the regular contracts, the primary physician is remunerated per consultation and treatment. In the case of GP-centred care, the GP receives an additional amount per capita directly from the insurance company.

The federal state of Baden-Württemberg is one of Germany’s larger states with about 10 million inhabitants. It is organized into several *Landkreise* (counties) with variable population density, ranging from 3008 inhabitants per km^2^ in the urban region of Stuttgart to 101 inhabitants per km^2^ in the rural Main-Tauber County [25]. Likewise, the number of dermatologists per inhabitant varies from one statutory health insurance (SHI) dermatologist per 9617 inhabitants (City of Karlsruhe) to one dermatologist per 130,772 inhabitants (Sigmaringen County) [26]. The SHI GP-to-inhabitant ratio shows less variation: there is between one GP per 1070 inhabitants (Main-Tauber County) and one GP per 1501 inhabitants (Tuttlingen County) [26].

Eight counties were included in the study. Selection criteria for the counties were the closeness to the study center (Tübingen) and their similarity in study-relevant indicators (see Table 1). These parameters will be used for the counties’ matching and randomization process. Allocation of patients to control or intervention groups (−t_2_ in Fig. 1) is based on this randomization.

**Table 1:** Matching parameters of the eight study counties in the federal state of Baden-Württemberg

County	Inhabitants per km^2^	Inhabitants	Dermatologist:inhabitant ratio	GP:inhabitant ratio
Böblingen	617	381,281	1:25,419	1:1352
Calw	195	155,359	1:51,786	1:1387
Esslingen	817	524,127	1:29,118	1:1368
Freudenstadt	133	116,233	1:58,117	1:1471
Reutlingen	274	282,113	1:18,808	1:1190
Rottweil	179	137,500	1:27,500	1:1127
Tuttlingen	186	136,606	1:27,321	1:1501
Zollernalbkreis	206	188,595	1:31,433	1:1266

*GP* general practitioner

### Study design

During the first phase of the study (−t_1_ in Fig. 1), GP teams will be recruited in the intervention counties. Further preparations will include the preparation of data protection policies and legal negotiations between insurance companies, middleware providers and GPs. Next, a run-in phase of 6 months will commence. In this phase, the teledermatology system will be implemented in the GP practices and GPs will be trained in the use of the system.

**Fig. 2:**
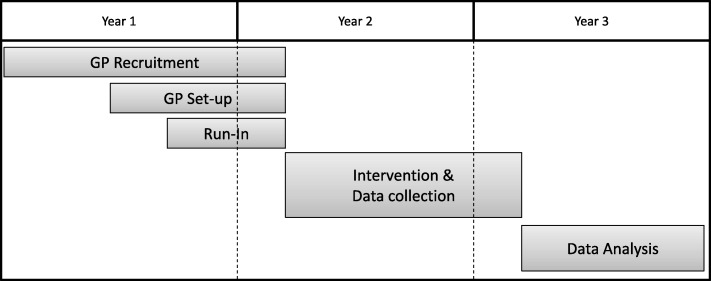
Project timeline of the 3-year project. GP general practitioner

After the run-in phase, 14 months after the beginning of the study, the intervention phase will start. From this point onward, patients can be enrolled (t_0_ in Fig. 1). The phase will last 1 year. Then, a 10-month analysis and publication phase will follow. See Fig. 2 for an overview.

**Fig. 3:**
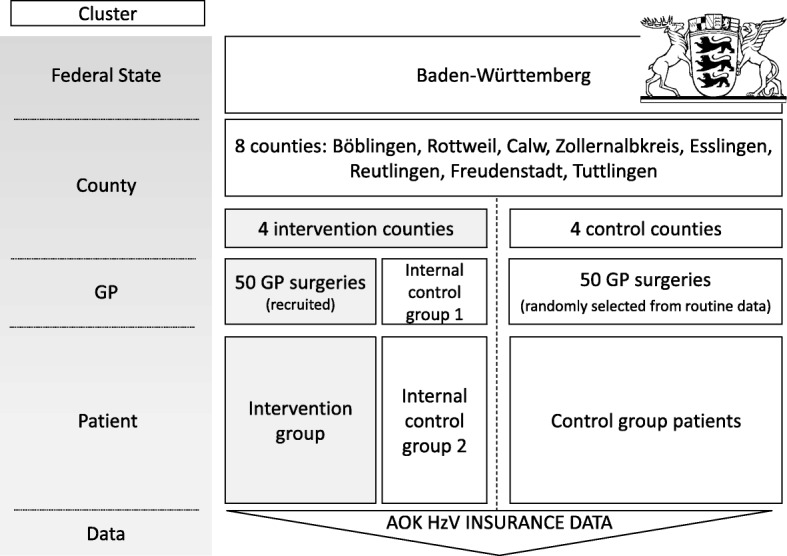
Overview of clusters: different levels or clusters of the project. AOK Allgemeine Ortskrankenkasse, GP general practitioner, HzV Hausarztzentrierte Versorgung

The cluster level “county” is randomized into four intervention and four control counties. In the intervention counties, data from non-participating HzV practices will serve as an internal control group at the cluster level “practice”. At the cluster level “patient”, data from nonparticipating patients are used as a second internal control group. No blinding is applied to any level of the trial. Figure 3 shows both study arms and an overview of the different cluster levels.

The Institute of General Practice and Interprofessional Care acts as the coordinating center and project lead. In collaboration with the AOK, aQua and the Institute for Clinical Epidemiology and Applied Biometry (IkEaB), the institute also acts as the data management team.

### Outcomes

The primary outcome measure is the number of conventional referrals from GPs to dermatologists.

Secondary endpoints will be evaluated using a mixed-methods approach. This will allow us to evaluate different aspects of the implementation process. For instance, the teledermatology system will provide information about the referral time and process quality. This data source is complemented by questionnaire surveys from all participating groups. Both care providers (GPs, dermatologists, GP practice staff) and patients (teleconsultation patients and patients who have declined a teleconsultations) will receive questionnaires on acceptance and feasibility. Patient questionnaires will also include all items from the Dermatology Life Quality Index (DLQI) [27]. Semi-structured face-to-face interviews (*n* = 15 patients who have received a teledermatology referral and n = 15 care providers, such as GPs, dermatologists and GP practice staff) will provide additional information to the surveys. Lastly, a health economics analysis based on routine data and support costs for the implementation of the teledermatology system is planned. Study sites (GP practices) will be visited once during the intervention phase by a study nurse for audit.

Details about the outcome parameters are presented in Table 2.

**Table 2:** Primary and secondary endpoints, data sources and evaluation methods

Secondary endpoints (intervention group)					Primary endpoint (both groups)
Patients	GPs	Dermatologists	GP team staff	Teledermatology software/KSYOS (process evaluation)	Routine data
Care provider/customer satisfaction, description of technical processes with their advantages and disadvantages				• Number of teleconsultations • Time until dermatologist answers • Number of physical referral recommendations • Number of queries by dermatologists after teleconsultation • Type of disease treated by teleconsultation • Duration of complete teleconsultation process • Result of dermatologic consultation	• Number of referrals to dermatologists by GPs • Ambulatory dermatologic diagnoses • Ambulatory patient contacts with GPs and/or dermatologists due to skin conditions (including EBM^a^ keys) • Number of clinic referrals due to skin conditions
• Quality of life (DLQI, questionnaire) • Satisfaction with dermatologic care/telemedicine (questionnaire, interviews)	Appraisal of feasibility, practicability and barriers for implementation (interviews)				
	• Number of reports by dermatologists after consultation (KSYOS and GP PMS) • Time until dermatologist report completed (PMS)	• Recommendation of physical referrals by teledermatologists			

*DLQI* Dermatology Life Quality Index, *EBM* Einheitlicher Bewertungsmaßstab, *GP* general practitioner, *KSYOS* teledermatology system, *PMS* patient management software

^a^Physician’s fee table used to encode what kind of procedure is performed in an outpatient setting and how much money will be paid for it; data are transferred from a practice to the Kassenärztliche Vereinigung and then generate a defined amount of income for the physician

### Intervention (t_1_ in Fig. 1)

The intervention is the provision of all skills and equipment needed to use teledermatology in general practice. If a GP does not feel secure about the management of a dermatologic case, he or she can use teledermatology. Initially, the GP takes standardized pictures of the lesion with a digital camera (Sony Cyber-shot DSC-W810). Next, high-resolution close-up pictures of the lesion are made using a polarized dermatoscope (MEDL4DW DermaScope Polarizer). The GP then creates a teleconsultation. Case-relevant patient data from the patient management system (PMS) are automatically included using middleware. If needed, the GP may provide additional case-relevant information such as the patient’s medical history and a description of symptoms. This information is then pseudonymized and transferred to a server in the Netherlands (KSYOS teledermatology system [3]). Dermatologists with teledermatology training are then notified. If possible, the dermatologists diagnose the lesion and recommend an appropriate course of action. The thus completed report is then sent back to the GP within 48 h. Both the data transfer and authentication use Secure Hypertext Transfer Protocol (https) connections with 256-bit Secure Hash Algorithm (SHA) encryption. An encrypted digital certificate is needed for authentication.

If the information provided by the GP proves insufficient, the dermatologist may either recommend a physical referral to a dermatologist or initiate a “second round”, in which the GP resends the case with possibly better picture quality and more information on the case.

If malignant lesions are found, patients will be referred to specialized dermatologic care. No other restrictions concerning patient care are imposed on the GP teams.

### Recruitment and study flow

**Fig. 4:**
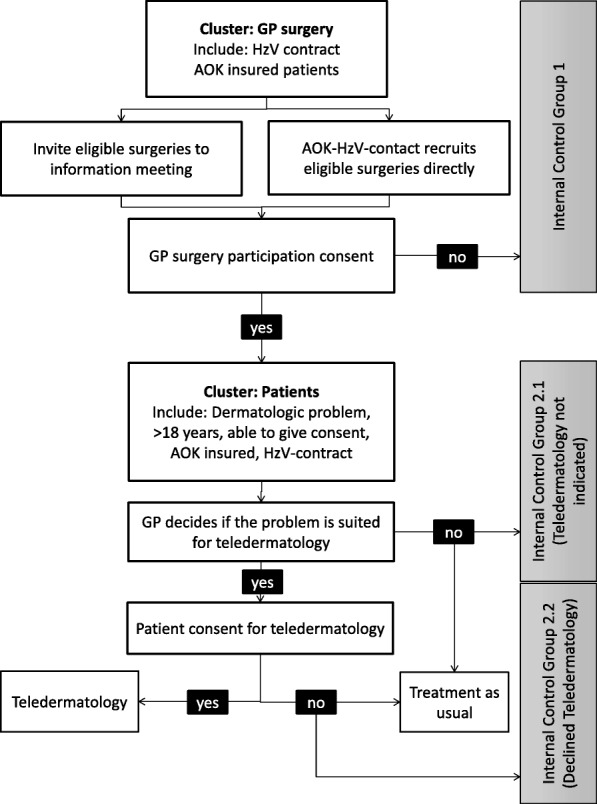
Recruitment and flow. Flow diagram (adapted and modified from the CONSORT statement) demonstrating how the two-step recruitment process and patient flow work. AOK Allgemeine Ortskrankenkasse, CONSORT Consolidated Standards of Reporting Trials, GP general practitioner, HzV Hausarztzentrierte Versorgung

The study contains a two-step enrolment process. The first step is to recruit eligible GPs in the intervention counties. Only HzV-contracted GPs will be included. The GPs and their teams will then in turn recruit study participants during the intervention phase (t_0_ in Fig. 1, start 1 July 2018). The patient population of this study comprises all HzV-enrolled, AOK-insured patients in the eight counties. Patients must be at least 18 years of age. Detailed inclusion and exclusion criteria and their role in the recruitment process are shown in Fig. 4.

Study participants are allocated as follows. The family doctors inform eligible patients (see Fig. 4 for criteria) about the study and ask their written consent to participate. After consent has been given and the teledermatology process has been completed, the family doctor enters a value in the patient's PMS entry. This value is visible in the subsequent data analysis and identifies the patient as a study participant. Routine data from the general practitioner's practice PMS are then transmitted to the AOK quarterly via a secure data connection. The AOK aggregates the data for the intervention and control circles.

No written consent is required from patients in the control groups. When patients sign an HzV contract with their insurance company, they consent to the anonymized analysis of their insurance data for scientific and quality management purposes.

Candidates in the intervention group practices who do not meet inclusion criteria (e.g., patients insured by other statutory health insurances or not listed as HzV patients) as well as patients refusing the intervention will be treated as usual and referred to a dermatologist. Eligible patients refusing the interventions will be included in an internal control group (see Fig. 4).

### Sample size calculation

The main scientific hypothesis is that the possibility of teledermatology in general practice will reduce the number of referrals to a dermatologist by 15% in the intervention group. The statistical null hypothesis is a reduction of 0%, the alternative is a reduction > 0%. The basis of the sample size calculation is an assumed reduction of 15%.

A mean of 120 referrals to dermatologists per year and GP is assumed. In 2015, 40% of AOK-insured patients were enrolled in the HzV [28, 29]. Thus, for the subgroup of AOK patients, 48 dermatological referrals per year and GP practice are to be expected in the control population during the 1-year intervention phase of the study. A Poisson distribution of the referrals for each GP team is assumed. Furthermore, a type I error of 5% (two-sided) and a type II error of 20% are defined. These assumptions lead to a calculation of 36 analyzable GP teams (with, on average, 48 referrals per year and GP practice each) per study arm. On the patient level, this amounts to 1728 patients with the indication for a referral to a dermatologist in total. A 15% reduction equals 260 referrals in total. A 30% dropout buffer is taken into account. The final sample size calculation amounts to 2400 patients with a reduction of 360 referrals.

### Data analysis (t_2_ in Fig. 1)

The pooled routine dataset is sent from AOK to the aQua institute. The aQua institute selects, filters and anonymizes the data. Lastly, the aQua institute delivers the anonymized patient dataset to the Institute for Clinical Epidemiology and Applied Biometry (IKEaB) for statistical analysis. All of these transfers use a secure, encrypted protocol and are authenticated. The relevant data sources are subject to extensive quality management processes. To secure data protection of interview and questionnaire data as well as process data from the KSYOS system, the Tübingen University Hospital’s (UKT) data protection agency is involved. They are independent from the sponsor.

For the primary outcome, Poisson regression is chosen as an analysis tool. To consider practice size and patient load, the number of practice KV bills during the intervention phase is applied as an offset. The dichotomous factor “study arm” is applied for the calculation. The GPs are the unit of observation. For both study arms, separate confidence intervals are created for the number of referrals per KV bill.

For the sample size calculation, a simple *t* test was used. In contrast to the sample size calculation, the statistical analysis will use a more complex method (Poisson regression). This is expected to lead to an increased power of the study, because the offset “total number of KV bills during intervention” explains parts of the variability between GP teams. Study power should thus be over 80%. Primary analysis will be calculated as a Poisson regression with a significance level of 5% (two-sided). Analogous methods will be applied to secondary outcomes (see later). However, the interpretation of local significances is not designed to be strictly confirmatory. Due to the multitude of secondary parameters lacking hierarchical structure, a correction for multiple tests is unrealistic. Descriptive analyses are performed following scaling and observed data distribution.

Secondary outcomes are analyzed using adequate regression models (linear, ordinal, logistic, multinominal) and considering cluster effects. The generalized estimating equations (GEE) method is employed. For GP team-related analyses (unit of observation = GP), appropriate regression models without cluster adjustment are used, because the outcome is only measured once per GP team. This is especially true for analyses based on KSYOS data (process data) and AOK insurance data. The same applies for secondary endpoint analyses among dermatologists, GPs and nonphysician staff. Questionnaires will be piloted using the “think aloud” method [30]. During the pilot, some participants (patients and care providers) are asked to read questionnaire items and voice their thoughts on them. A research team member observes the participants and creates a protocol. The protocols of these observations are then used to improve the questionnaire.

Interviews are transcribed and then analyzed using qualitative content analysis [31]. Analysis will continue until thematic saturation is reached. The resulting category system will provide information additional to the questionnaire data and help understand patients’ and care providers’ perspectives on teledermatology.

Ancillary studies may include analysis of anonymized process data and/or anonymized pictures of lesions. Design and planning will be presented in separate publications.

### Health economic analysis

Additionally, a health economic analysis will be conducted (t_2_ in Fig. 1). The evaluation shall provide an estimate of the marginal cost advantage of teledermatology referrals compared to conventional referrals. Furthermore, conclusions may be drawn as to which parameters offer particularly high marginal cost advantages. This is of crucial importance, particularly with regard to the transferability of such interventions to other specific fields and regions.

Under the assumption of comparable effects between teledermatology and conventional referrals, a cost minimization analysis will be pursued for health economic evaluation [32]. For this purpose, the quantity structure of costs and the costs of the teledermatology intervention as well as the conventional treatment will be constructed. The health economic evaluation will consider two cost perspectives separately. In the first scenario, only the costs incurred to the SHI will be accounted for; while in the second scenario, the societal costs will be examined to illustrate the cost difference for the SHI and the society as a whole. The costs of implementation, treatment and diagnostics for GPs and dermatologists will be considered in the cost minimization analysis of both scenarios. In the analysis from an overall social perspective, the travel times and costs to patients will additionally be taken into account. The routine data of AOK Baden-Württemberg will serve as the data source for the evaluation. Furthermore, costs related to the intervention, such as the costs of technical equipment and training, will be collected and analyzed.

## Discussion

We present the study protocol of a cluster-randomized clinical trial aiming at implementing teledermatology into general practice in Germany. Internationally, teledermatology is established in general practice as a safe and cost-effective way to treat patients with dermatologic problems. Cost savings and benefits for patients are especially pronounced in rural areas with long distances to dermatologists. So far, only a few projects using store-and-forward technology have tried to establish teledermatology in German health care. None of these projects have transferred into routine care. There are numerous reasons that contribute to the difficulties in implementing telemedicine in general in the German health care system. Understanding their implications for our study and considering them in planning and conducting the study is thus important for its success.

One reason contributing to the difficulties in implementing telemedicine is the rather restrictive data protection laws in Germany. Data protection and ownership are culturally valued topics in Germany. Thus, German data protection laws guarantee high data safety for patients, while providing little room for innovation, even in scientific settings or pilot studies. This is evident when comparing the German situation to the Netherlands or the Scandinavian countries, where a more centralistic and less restrictive data protection culture prevails. GPs and patients might therefore choose not to participate or to drop out of the study due to data safety concerns. In our study, we provide information to all participants about data safety and protection. All study partners have agreed on a data protection policy that follows EU regulations.

Another challenge for the implementation of telemedicine in German primary care is the heterogeneity of practices. Basically each aspect of daily work (team structure, patient management software, size of the practice, practice organization) is different between practices [33]. For example, about 235 different digital patient data management systems exist in Germany [34]. In 2017, German law enforced the implementation of a generic patient data interface within 2 years [35]. Using such an interface is currently the only way to export patient data to third-party solutions, like the KSYOS teledermatology system. Nevertheless, there still are some PMSs that do not support such an interface or ask for licensing and support fees. The GP may use the teledermatology nevertheless by manually importing and exporting data, but that might prove too time-consuming for daily practice. The consequence might be that the GP drops out of the study.

To ensure the intervention’s success, GPs must be allowed to choose their own way of implementing the intervention in their practice. Thus, it is allowed for GPs to delegate parts of the teledermatology procedure (e.g., photography of the skin lesions) to practice staff. This way, the acceptance of the intervention hopefully increases, thus resulting in more realistic and reliable results.

In order to grasp the multitude of different perspectives, a mixed-methods evaluation was designed. We hope to identify relevant barriers and enablers for the implementation of sustainable teledermatology in routine general practice. If we succeed, the TeleDerm study could be a milestone in the implementation of telemedicine in general and teledermatology in particular in Germany.

### Strengths and limitations

The heterogeneity of GP practices, as stated earlier, is one of the major challenges of the study. We expect this to have an impact on the activity of the referring GPs. Therefore, we will recruit 30% more GPs than needed for statistical power to suffice. The internal control group allows us to examine cluster effects.

During the run-in phase of 6 months, first experiences with the technical implementation and the application of the teledermatology system in daily practice are made. This information will allow adjustments of the implementation processes in the GP practices. Also, we aim to address possible data safety concerns on all sides (GPs, patients, AOK). Experiences collected during run-in will be used to constantly inform and improve the ongoing implementation process.

An advantage of this study is that GP participants need little extra training. The intervention is tailored and very close to the reality of routine general practice in Germany. This, with the gained experiences from the study, will help to facilitate a bigger roll-out of teledermatology in German general practice in the future. To enable and plan a large-scale roll-out is a prerequisite for the funding for this study.

Our design allows us to collect information that has an impact from the implementation into routine care such as acceptance among patients and GPs. If the study succeeds, it will be the first teledermatology store-and-forward project to actually pass the barrier from a research project into daily practice, thus reaching new horizons for the much anticipated advance of eHealth in Germany.

### Trial status

At the time of manuscript submission, the study design has been evaluated by an independent international reviewer and has been approved by the responsible ethics committee of the UKT. Recruitment and enrolment of patients started in July 2018.

### Protocol version and history

Current: Version 2, 9 August 2017. Past: Version 1, 16 June 2017.

## Additional file

Additional file 1: SPIRIT 2013 checklist. (DOC 123 kb)

## Abbreviations

AOK: Allgemeine Ortskrankenkasse
GEE: Generalized estimating equations
GP: General practitioner
HzV: Hausarztzentrierte Versorgung
IKEaB: Institut für Klinische Epidemiologie und angewandte Biometrie
KV: Kassenärztliche Vereinigung
PMS: Patient management software
SHI: Statutory health insurance
UKT: University Hospital Tübingen
